# Pectin Oligosaccharides Ameliorate Colon Cancer by Regulating Oxidative Stress- and Inflammation-Activated Signaling Pathways

**DOI:** 10.3389/fimmu.2018.01504

**Published:** 2018-06-27

**Authors:** Haidong Tan, Wei Chen, Qishun Liu, Guojun Yang, Kuikui Li

**Affiliations:** Liaoning Provincial Key Laboratory of Carbohydrates, Dalian Institute of Chemical Physics, Chinese Academy of Sciences, Dalian, China

**Keywords:** immunomodulation, colon cancer, pectin oligosaccharides, signaling pathway, prebiotics

## Abstract

Colon cancer (CC) is the third common neoplasm worldwide, and it is still a big challenge for exploring new effective medicine for treating CC. Natural product promoting human health has become a hot topic and attracted many researchers recently. Pectin, a complex polysaccharide in plant cell wall, mainly consists of four major types of polysaccharides: homogalacturonan, xylogalacturonan, rhamnogalacturonan I and II, all of which can be degraded into various pectin oligosaccharides (POS) and may provide abundant resource for exploring potential anticancer drugs. POS have been regarded as a novel class of potential functional food with multiple health-promoting properties. POS have antibacterial activities against some aggressive and recurrent bacterial infection and exert beneficial immunomodulation for controlling CC risk. However, the molecular functional role of POS in the prevention of CC risk and progression remains doubtful. The review focuses on antioxidant and anti-inflammatory roles of POS for promoting human health by regulating some potential oxidative and inflammation-activated pathways, such as ATP-activated protein kinase (AMPK), nuclear factor erythroid-2-related factor-2 (Nrf2), and nuclear factor-κB (NF-κB) pathways. The activation of these signaling pathways increases the antioxidant and antiinflammatory activities, which will result in the apoptosis of CC cells or in the prevention of CC risk and progression. Thus, POS may inhibit CC development by affecting antioxidant and antiinflammatory signaling pathways AMPK, Nrf2, and NF-κB. However, POS also can activate signal transduction and transcriptional activator 1 and 3 signaling pathway, which will reduce antioxidant and anti-inflammatory properties and promote CC progression. Specific structural and structurally modified POS may be associated with their functions and should be deeply explored in the future. The present review paper lacks the important information for the linkage between the specific structure of POS and its function. To further explore the effects of prebiotic potential of POS and their derivatives on human immunomodulation in the prevention of CC, the specific POS with a certain degree of polymerization or purified polymers are highly demanded to be performed in clinical practice.

## Introduction

Colon cancer (CC) is one of the third common cancers with more than 600,000 deaths worldwide yearly and causes a global burden (1). Chemotherapy and radiation therapy are the main treatments of CC with significant side effects. A dietary prebiotic improves glycemic indices, lipid profile (2, 3), antioxidant status (4), potential immunomodulatory benefits (5), and reduces cardiovascular disease risk (6). The common prebiotics are oligosaccharides while oligosaccharides are indigestible and pass through digestive tracts smoothly. The oligosaccharides produced in digestive tracts will promote the production of volatile fatty acids, which can release constipation, reduce serum blood glucose, improve mineral absorption and lipid metabolism, prevent colonic cancer, inhibit pathogen adhesion, and modulate immune activity. Pectin oligosaccharides (POS) belong to new potential prebiotics with various health-promoting effects (7, 8), such as against Shiga toxins (9) and pathogen binding (10), induction of apoptosis of human colonic adenocarcinoma cells (11), immunomodulation (12, 13), and cardiovascular protection (14). Long-term pectin consumption has been found to suppress weight gain and reduce obesity risk in an animal obesity model (15). Pectin is an efficient medication to repair wounds and an effective prophylaxis during surgery with antibacterial activities (16). POS exert antioxidant, anti-inflammatory, and antinociceptive effects. Grapefruit pectin (*Citrus paradisi*) can improve lipid profiles (17). In addition, POS are safe and non-mutagenic, and can be used in children food (18, 19).

Pectin oligosaccharides can stimulate apoptosis process in human colonic adenocarcinoma cells, show protective functions for cardiovascular tissues, reduce the damage caused by metals, and have anti-obesity effects, antitoxic, antibacterial, and antioxidant activities (20). Sweet potato pectin possesses anticancer activity and induces the apoptosis of CC cells and may be a cancer therapeutic drug (21). The pectin derivative with maleoyl groups also shows antitumor properties for CC (22).

Pectin oligosaccharides have also been used to treat gastrointestinal disorders (23), diabetes (24), and hypercholesterolemia (25). Specifically, POS consumption can increase probiotic flora in gastrointestinal tract, such as *Lactobacillus Eubacterium, Faecalibacterium*, and *Roseburia* (26). Similarly, POS increase bifidobacteria population but no change in *Clostridium* (27). Arabinose oligosaccharides can be selectively used by *B. adolescentis, B. longum, B. vulgatus*, and *Lactobacillus* (28). POS promote the growth of bifidobacteria in all population from younger adults to the elders, and increase their immunomodulatory capacity (29) while the increase of immunomodulation further promotes the apoptosis of CC (30).

**Figure 1:**
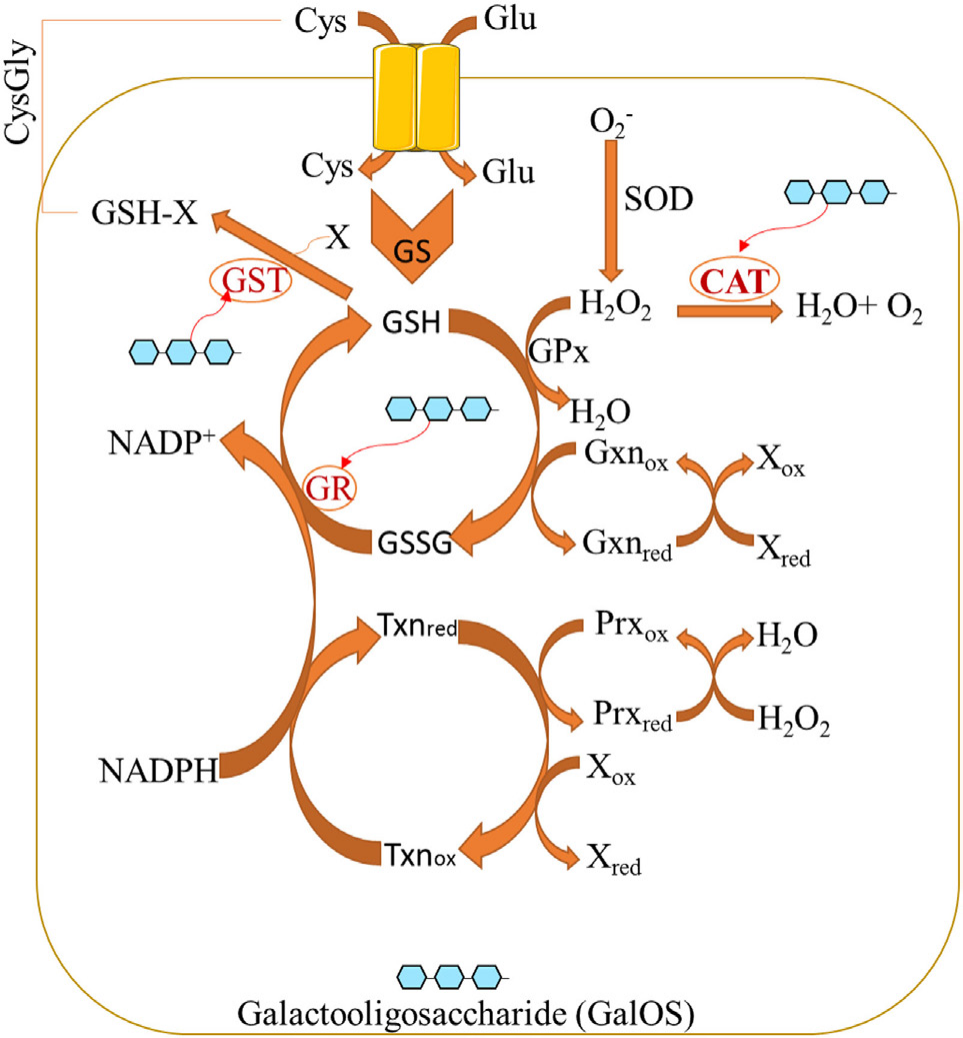
Pectin oligosaccharides regulate cellular antioxidant activities by affecting oxidative stress biomarkers.

Pectin oligosaccharides exert its antioxidant properties by significantly increasing the levels of antioxidant biomarkers while reducing oxidative biomarkers (31). The redox system may be regulated by POS (Figure 1). POS (as bioactive components of pectin) normalize the activity of glutathione reductase (GR) and glutathione peroxidase (GPx) (32), whereas GR catalyzes GSSG into reduced glutathione (GSH). GPx catalyzes H_2_O_2_ into H_2_O under the help from GSH. Furthermore, catalase (CAT) can be induced by POS (33) whereas CAT reduces H_2_O_2_ into H_2_O. POS also increase glutathione-*S*-transferase (GST) activity (31), while GST promotes the generation of plasma-reduced CysGly during GSH catabolism.

**Figure 2:**
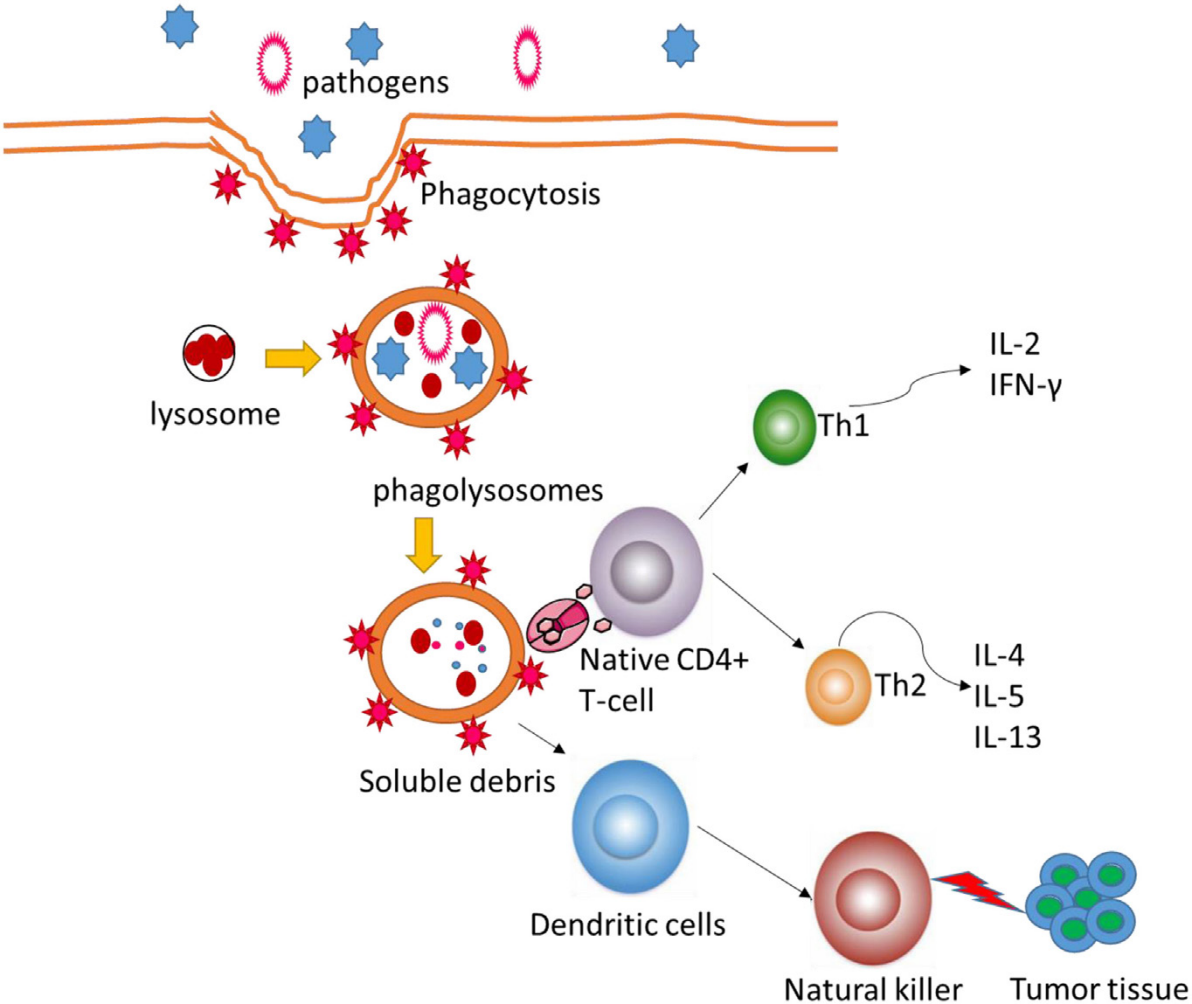
Pectin oligosaccharides regulate cellular autophagous activities by affecting natural killer.

The POS homogalacturonan (HG), isolated from green tea, shows phagocytosis-enhancing activity in HL-60 cells (34). Meanwhile, POS will increase natural killer bioactivity and the levels of anti-inflammatory cytokines (35) and reduce the levels of pro-inflammatory cytokines (Figure 2). POS can be developed as a beneficial dietary candidate for promoting gastrointestinal health and immune activities. Antioxidant and anti-inflammatory activities of functional foods will be beneficial in the prevention of the risk of colon carcinoma (36, 37). Nevertheless, the molecular mechanisms for POS function in human health remain doubtful. This work provides a new window for the possible effects of POS on antioxidant and anti-inflammatory signaling pathways.

## POS Preparation

### Pectin As a Source of POS

**Figure 3:**
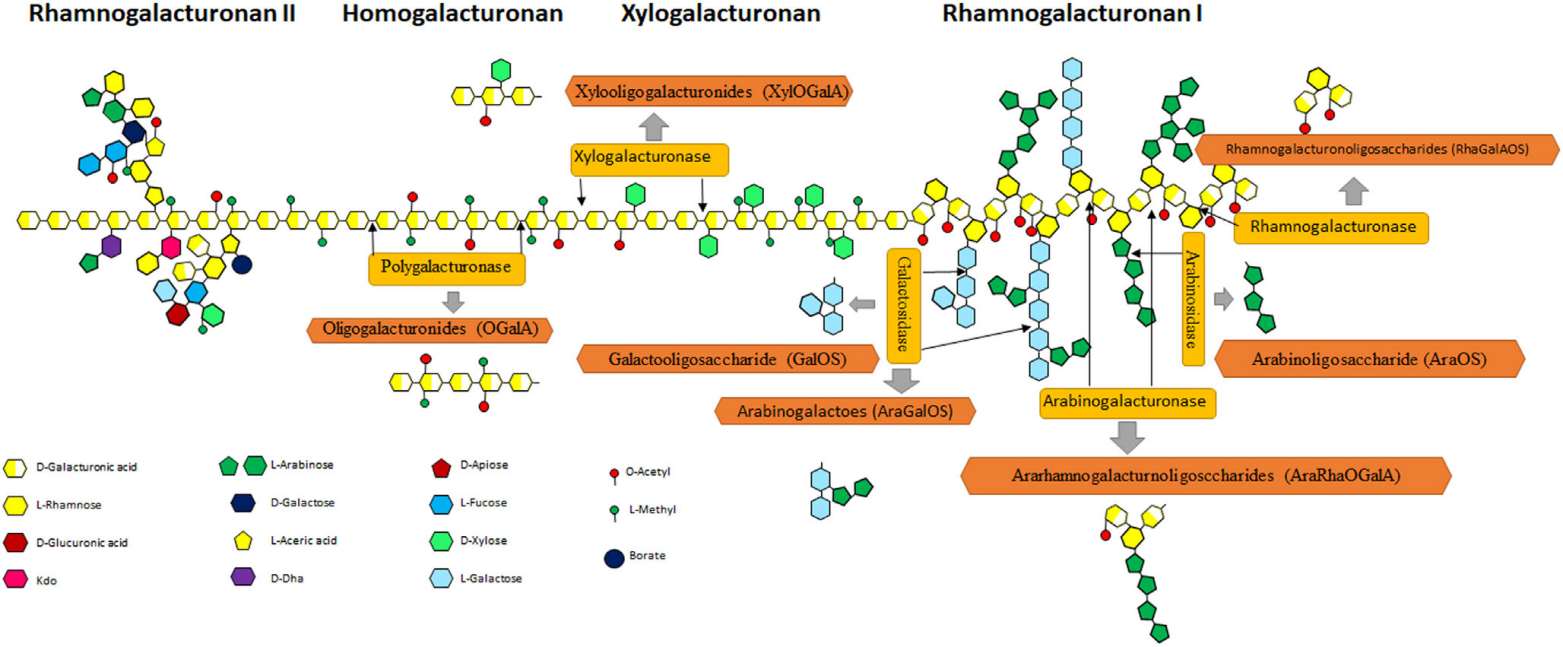
Schematic representation of pectin structure. Pectin consists of rhamnogalacturonan I (RG-I), homogalacturonan (HG), xylogalacturonan, and rhamnogalacturonan II regions. HG is a linear polymer consisting of a chain with an estimated length of 72–100 GalA units that represent, approximately, 60% of the total pectin (41). Xylogalacturonan is a chain of GalA residues partially substituted by d-xylose residues connected by β-(1,3) links at C-3 and/or C-2 positions. RG-I represents up to 7–14% of pectin and contains alternating units of α-(1,4)-galacturonosyl and α-(1,2)-rhamnosyl (42). In many cases, rhamnose residues show side chains as substituents on the O-4 position, made up of arabinan and/or arabinogalactan I and II, although xylose or glucose modification also exists (43). Rhamnogalacturonan II (RG-II) is a region characterized by a length of 7–9 GalA units, where complex branches made up of 12 types of monosaccharides (as a maximum) can exist, including some minority monomers such as apiose, fucose, acetic acid, DHA, or KDO (44).

Pectin is the source of POS in natural products and mainly exists in citrus peel [it mainly consists of a homopolymer of 1–34-linked os-d-galactosyluronic acid with 85.7% methylated esterification and a rhamnogalacturonan I (RG-I) fragment] (38), sugar beet pulp (a high degree of acetylation and a relatively high neutral sugar content) (39), potato pulp (it has highly branched RG-I domain) (21, 40), and additional sources, etc. Pectin consists of fundamental units of α (1–4)-galacturonic acid, which is often acetylated and/or methylated. Figure 3 shows the complex structure of pectin, consisting of HG, a polymer with free or esterified carboxyl group; rough regions consists of RG-I with some units of rhamnose and galacturonic acid; and rhamnogalacturonan II (RG-II) with galacturonic acid units and multiple modification. All these regions can be degraded into POS. Various POS can be produced from pectin *via* de-polymerization (Figure 3).

### POS Purification

Pectin oligosaccharides, as oligosaccharides, are often prepared by partial hydrolysis of pectin, which consists of complex heteropolysaccharides. There are three main methods for POS production, including bioenzymatic digestion (45), acid hydrolysis (46) or hydrothermal treatments, and high-pressure microfluidization (47). Many raw materials can be treated to obtain POS including orange, lemon, apple, beet pulp, and so on by using acids. There are some disadvantages for the chemical method: environmental contamination, simple products, and general toxicity. As an alternative, pectin can be degraded into peptic polymers by pectin enzymes. Although pectin has complex structures, which can be digested by a series of pectin enzymes, including hydrolases, lyase, and esterase (48–50). Since one enzyme generally targets only specific structure, and more defined oligosaccharides can be released when compared with chemical method. Finally, high-pressure microfluidization has been considered as a new method but most POS cannot be obtained by only using the physical techniques.

After production, purification processes are necessary to obtain food-grade final products. Membrane filtration is often used to purify specific POS. Diafiltration has been used to purify POS from the hydrolysis from lemon peel wastes and yields of target POS can reach 98 wt% of oligogalacturonides (2–18 DP) and AraOS (2–8 DP) (51). The similar work has been reported to achieve a refined POS with AraOS (3–21DP), GalOS (5–12 DP), and OGalA (2–12 DP) (52). Ultrafiltration and diafiltration have also been used to isolate AraOS, which can be further purified into specific POS by using a membrane with 1-kDa molecular weight cut-off (53). On the other hand, pectin can fulfill its function *via* its degraded products POS since pectin cannot be dissolved in water. In that case, POS are sometimes used to stand for pectin in subsequent introduction.

## POS Affect Mitogen-Activated Protein Kinases (MAPK) Signaling Pathway

**Figure 4:**
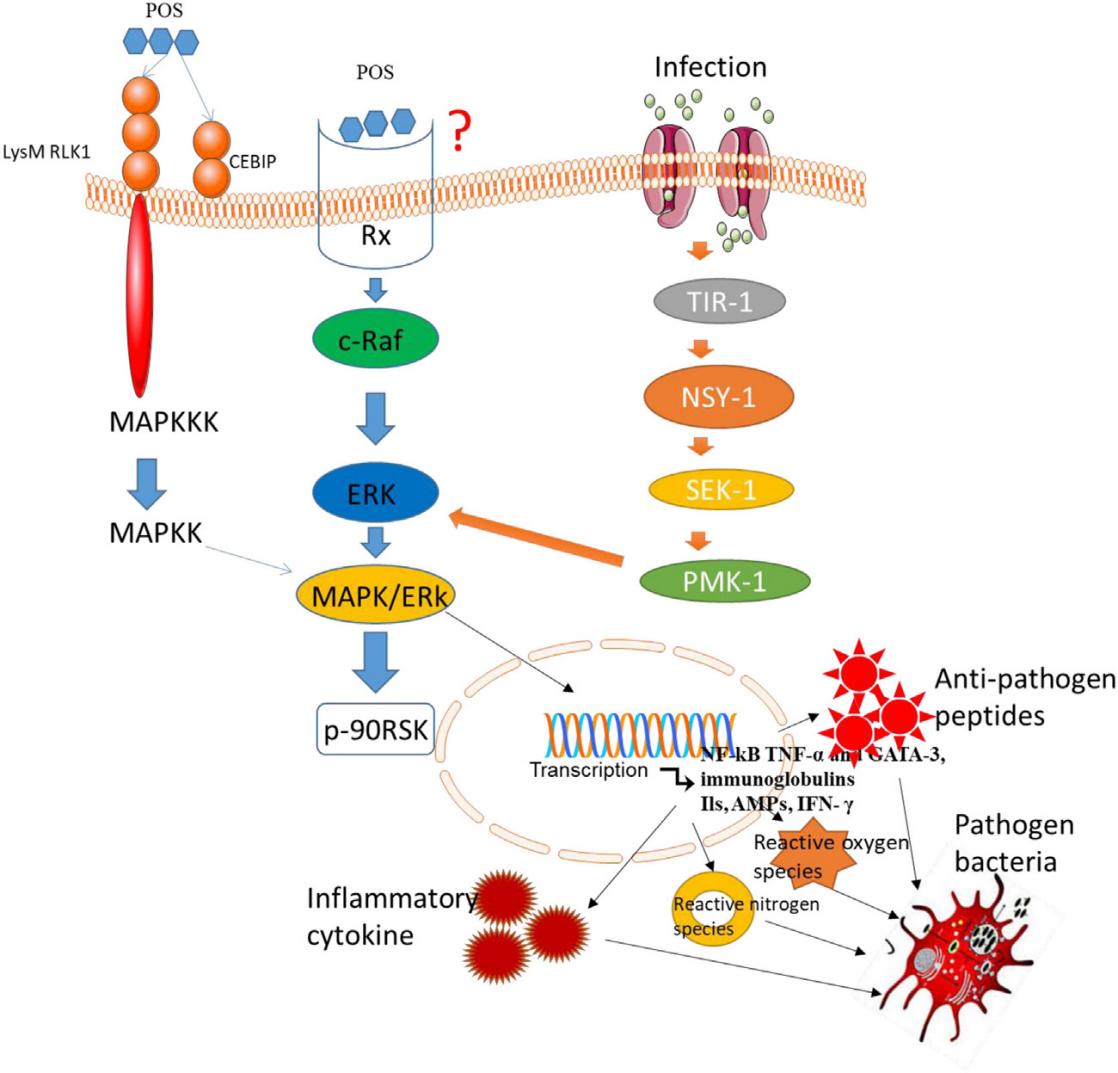
Pectin oligosaccharides binds potential membrane receptors in mitogen-activated protein kinases/ERK signaling pathway. LysM RLK1, chitin elicitor-binding protein, and RX are potential receptors in the pathway.

The MAPK signaling pathway plays an important role in most immune responses (54, 55). Downregulation of MAPK signaling pathway can inhibit the proliferation, invasion, and angiogenesis of CC (56), and promotes the apoptosis of CC (57). Larch Arabinogalactan (a kind of POS) has been reported to inhibit p38 phosphorylation in MAPK pathways (58). Thus, POS may prevent the risk or progression of CC by suppressing MAPK signaling pathway. However, there are still inverse reports for the effects of POS on MAPK/EKR signaling pathway. Mammalian cells respond to various extracellular stimuli by activating MAPK/extracellular signal-regulated kinase (ERK) signaling pathway. Typically, ERK activates phosphorylation events, which stimulate Ras gene after activating growth factor receptor. The activation of Rapidly Accelerated Fibrosarcoma (Raf) phosphorylates ERK. Some targets of ERK have been identified, such as p90RSK activation *via* Ser380 phosphorylation (59) (Figure 4). POS promotes the phosphorylation of ERK (60) and may also activate the phosphorylation of Raf, MEK, and p90RSK (Figure 4). Thus, POS may bind the receptor systems that activate Raf, MEK, and ERK since POS cannot transport across plasma membrane. ERK signaling pathways can be activated by POS, suggesting that there is an oligosaccharide receptor that transfers the information to the activated molecules (Figure 4). The final genetic identification of all components of the POS signals remains to be determined. Several evidence suggests that p90RSK is activated by MAPK (61). The activation of MAPK signaling pathway will increase antioxidant activities (62) properties. Furthermore, increasing antioxidant activity and activating MAPK signaling will result in the apoptosis of CC cells (63).

The lysin motif receptor-like kinase is necessary in the activation of chitin-induced signals (64). Furthermore, chitin elicitor-binding protein (CEBiP) has a LysM domain and is also a surface receptor for plant chitin (65). LysM domain-containing protein pectate lyase (66) suggests that POS has high affinity with LysM domain. Thus, LysM RLK1 and CEBiP may be potential receptors of POS (Figure 4). In general, POS binds potential membrane receptors and activates MAP3K, which activates MAP2K, resulting in the activation of MAPK, which can activate related transcription factors. Besides of these receptors, POS may interact with many membrane receptors. Capsaicin represents an important class of surface receptors (67, 68). Therefore, they cast light on how the cells regulate biological events.

### POS Regulate STAT 1 and 3 Signaling *via* Leptin Receptor

Signal transduction and transcriptional activator 1 (STAT1) is encoded by STAT1 gene in human being. Specific expression of STAT1 can be mediated by some cytokines, such as IFN-α (69, 70), IFN-γ (71, 72), or IL-6 (73, 74). IFN-α binds receptor and triggers STAT signal *via* its phosphorylation and activation of STAT1 and STAT2. STAT binds ISGF3G/IRF-9 and forms a complex, which stimulates IFN-3 and IFN-9. STAT1 plays a key role in gene expression, cell survival, viability, or response to pathogens. In response to IFN-γ stimulation, STATl forms a homodimer or heterodimer with STAT3. The activation of STAT1 will improve the antitumor capability for CC (75). STAT1 deletion will change the interactions between tumor and fibroblast cells and contribute to CC progression, suggesting that STAT1 is an important link between intestinal inflammation and CC (76). In contrast, the activation of STAT3 signaling pathway regulates the pathogenesis of colon tumor (77).

### Oxidative Stress and Inflammation Activates STAT 1/3 Pathways

STAT 1/3 signaling pathways participates in cellular responses to cytokines or growth factors. ROS activates STAT 1/3 pathways in the exterior membranes of basilar blood vessels (78). This pathway can cause morphological varies of the wall of blood vessels in brainy vasospasm (79). Oxidative stress is closely associated with the cell apoptosis and induces STAT activation (80). STAT1 and STAT3 inhibitors suppress TLR-induced TNF expression (81). Viral replication and inflammation are associated with STAT pathway. The result suggests that activation of STAT 1 and 3 signaling pathway will develop inflammation *via* the increase in IFN level. The inactivation of the STAT pathway can improve anti-inflammatory activities (82).

### POS Regulate STAT-1 and -3 Signaling Pathways and Anti-Inflammatory Cytokine Secretion

**Figure 5:**
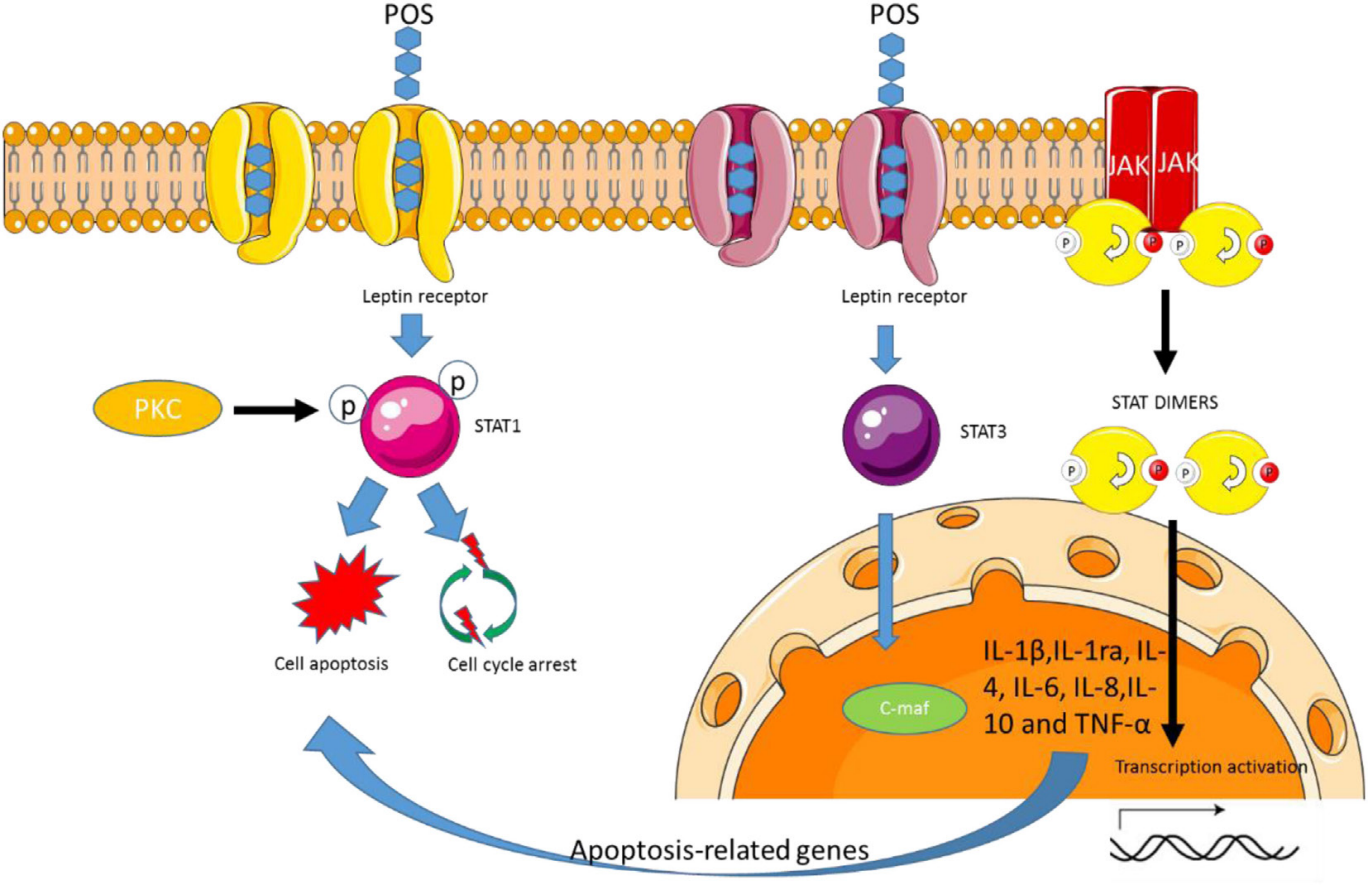
Pectin oligosaccharides (POS) regulates STAT 1 and 3 signaling pathway by the leptin receptor. POS-binding leptin receptor forms signal transduction and transcriptional activator 1 or STAT3 complex, which induces cell apoptosis or cell survival.

Pectin oligosaccharides promotes the expression of cardiotropin-1, which upregulates JAK and STAT pathway (14) and delivers the signals to cardiomyocytes, resulting in transcriptional, differentiating, and immune activity (Figure 5) (14). PKC is activated by a variety of agonists, including biological macrophage chemokines (83) and modulates a variety of allogeneic megakaryocytes (84). Pectin consumption will induce the expression of PKC (85), which promotes STAT1 phosphorylation (86). Thus, POS may modulate STAT1 activation and also depends on PKC (Figure 5).

Pectin can regulate biological activities *via* the interaction with immune cells. Pectin treatment increases TNF-α, IL-1β, and IL-10 cytokines (Figure 5) (87). Further work showed that the degree of methyl esterification, molecular size, and the characteristics of pectin structure were closely associated with the regulation of cytokine. These data suggest that POS variety will affect macrophages releasing chemokines. On the other hand, all the cytokines can be secreted by activating STAT signaling pathway (88). All the cytokines can be inhibited by preventing the activity of STAT pathway in macrophages (89). Thus, POS may affect the release of cytokines by regulating STAT signaling pathway (Figure 5).

Pectin oligosaccharides treatment promotes IL-1ra and IL-10 secretion (90), which may be beneficial to cartilage reparation. IL-1ra can inhibit the activity of IL-1β, whereas IL-1β overexpression is associated with osteoarthritis progression (91). Thus, the release of IL-1ra by POS-stimulated may help to protect the synthetic metabolic environment of the natural cartilage during bone cartilage repair. POS activating STAT-1 and -3 signaling pathways will not be beneficial to CC control while the increase of anti-inflammatory cytokines will result in the prevention of CC (37, 92).

### The Binding Between POS and Leptin Receptor

Pectin oligosaccharides has been regarded to have anti-obesity activities (15, 93). POS consumption increases leptin levels in adipose cells when compared to those without the treatment (*P* < 0.05). POS exerts anti-obesity properties *via* regulating appetite and satiety signals (94). An earlier report shows that POS can significantly decrease lipid accumulation by affecting lipid metabolism (95). POS from Hawthorn can reduce the concentrations of peroxisome proliferator-activated receptor γ, an important adipogenic regulating element (96). The POS tends to enhance TC level and to decrease sterol regulatory element-binding protein 2 and LDL receptor, suggesting that POS can be developed as a kind of functional food in improving lipid metabolism. Long-term pectin consumption can remarkably reduce lipid contents and decrease insulin and leptin resistance (97). Pectin diets can also reduce plasma leptin significantly by more than 60% in an obesity animal model (98). Leptin receptor (OB-R) can induce cardiac disorders (99) and also is linked with obesity development, which leads to obesity risk (100–102). Therefore, POS may affect these molecules by binding OB-R (Figure 5). Leptin regulates weight hemostasis (103, 104), reproduction (105), and possible hematopoiesis (106). Leptin receptor (OB-R) is produced in some alternating chunks of rodents (107) and humans (108). The activated JAK tyrosine kinase binds to ligands for rapid phosphorylation of STATS *via* the cytokine family of receptors (109). Gene transcription can be initiated by activating STATS homologous or heterologous fusion and migration to nuclear-binding STAT response elements such as GAS (IFN-gamma activation site). POS binding OB-R promotes the complex formation of STAT-1/3 (Figure 5). The low-level OB-R can activate STAT signal transduction pathway.

## POS Regulates Nuclear Factor-Kappa B (NF-κB) Pathway *via* Toll-Like Receptor

### POS Prevents Colonic Inflammation

The relationship between chronic intestinal inflammation and cancer has been widely reported (110, 111). The effect of POS on oral administration of colitis has been assessed by weight loss (112), disease activity index (DAI) (113), and bloody diarrhea events (114). DAI is associated with fecal consistency, fecal occult blood and weight loss. POS treatment significantly inhibits dextran sulfate sodium (DSS)-induced DAI (115). In addition, colon size is inversely proportional to the severity of DSS-induced colitis. These data indicate that POS can reduce intestinal inflammation in colitis mice. However, the related molecular mechanism remains widely unknown.

### NF-κB Signaling Pathway Is Involved With Inflammatory and CC

Nuclear factor-kappa B regulates DNA transcription, cytokine generation, and cellular life activities. NF-κB is existed in most animal cells types and involves the responses to cytokines, ROS, bacterial and viral antigens (116). Regulation of NF-κB is closely associated with CC (117, 118), inflammation and autoimmune disorders (119), septic shock, viral infection, and dysfunctional immunological progression (120). NF-κB can be affected by cellular antioxidant activities. The ratio of GSSG/GSH can strongly affect NF-κB pathway (121). NF-κB is linked with diabetic neuropathy and promulgation of inflammatory activity (122). The signaling pathway has protective functions for neuroinflammation and oxidative stress. NF-κB can affect brain edema and infarct volume, and its expression will result in inflammatory response after cerebral ischemia–reperfusion (123).

### POS Regulates NF-κB/TLR4/COX-2 Signaling Cascade

Ulcerative colitis (UC) is one common inflammatory bowel disorder and has high morbidity and prevalence throughout the world. UC is the main risk factor inducing CC (124). In UC patients, CC risk is higher than the average population (125, 126). The main feature of UC is the uncontrolled inflammation of the colon, causing acute abdominal pain, severe diarrhea, bloody stools, and reduced symptoms. The initiation and maintenance of colonic inflammation is characterized by the transmembrane invasion of leukocytes in the mucosa, the overproduction of inflammatory cytokines, etc., which are necessary for subsequent mucosal rupture and ulcers and involve in UC development, particularly in the early stages of disease (127, 128). Thus, UC therapy is mainly dependent on the drugs, which can inhibit colon inflammation and control symptoms.

However, conventional anti-inflammatory drug compounds generally have undesirable side effects, which may reduce patient compliance and degrade the condition. 5-aminosalicylic acid compounds and salazosulfa pyridine is considered first-line therapy for active UC therapy. However, side effects including abdominal pain, fever, diarrhea, cramps, rashes, and kidney failure limit their use. The lack of satisfactory treatment of UC has contributed to the study of alternative treatment strategies. Anti-inflammatory natural products or functional food from supplemental or alternative medicine represent a new class of drugs that are promising to UC therapy. Previous studies *in vitro* have found that POS can significantly and reliably attenuate lipopolysaccharide-induced inflammatory responses (129), demonstrating the potential medical utility of POS in controlling bowel disorder (130). The effect of oral POS on the prevention of inflammation has been proved, which shows a decrease in histological damage score and colonic PGE2 content in the mice with UC model and further confirmed the potential of POS for colitis therapy.

Apple POS has been proved to be effective to treat inflammatory and cancer diseases by affecting LPS/TLR4/NF-κB pathway (131). POS exerts beneficial effects on clinical colitis and carcinogenesis. Apple POS exhibit higher antibacterial effects on some pathogens than citrus POS (132). Staphylococcus has been reported to be isolated from the blood of the patients with cardiac disorder (133). The lipopolysaccharide derived from *Escherichia coli* and *Pseudomonas aeruginosa* induces cardiovascular damage (134). Apple POS prevent colon carcinogenesis that may partially depend on prostaglandin E, and POS types, which are associated with fecal enzyme function.

**Figure 6:**
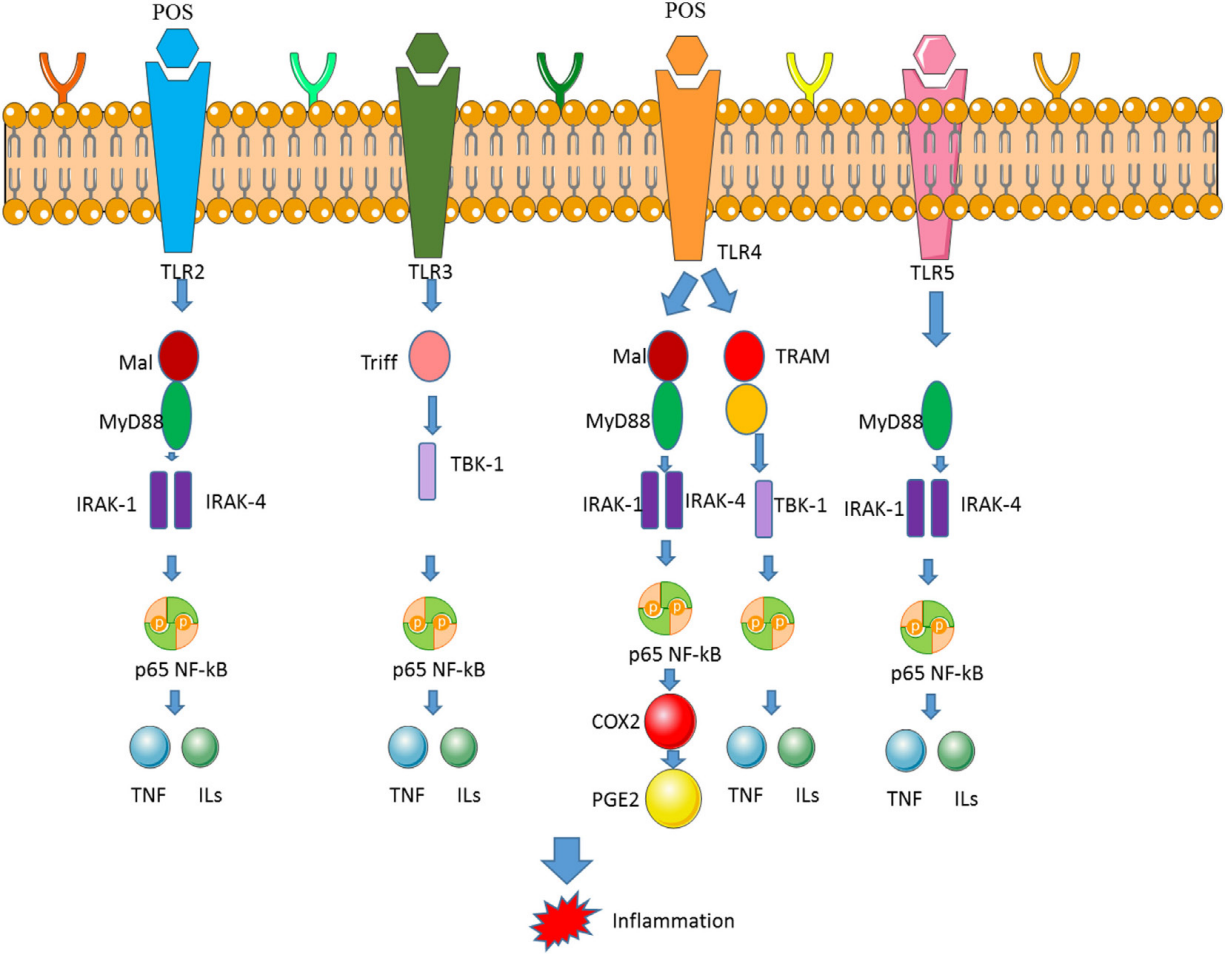
Pectin oligosaccharides (POS) downregulates the expression of nuclear factor-kappa B (NF-κB) and COX-2 by binding toll-like receptor 4 (TLR4). The binding between TLR4 and POS activates NF-κB and COX-2 signaling pathway, which is associated with inflammatory activities.

Apple POS can modulate inflammatory activities by affecting NF-κB pathway (131). Normally, NF-κB forms a p65-p50 dimer, which enters into the nucleus and binds specific DNA sequence, and inhibits target gene expression. POS may inactivate NF-κB and affects the level of its downstream genes [cyclin D1 (135), TNF-α (136), and IL-6 (115)] have been tested in NF-κB signaling pathway. Some data show that POS are the most potent activators of NF-κB signaling (Figure 6) (137), whereas the activation of NF-κB signaling pathway will promote CC apoptosis (138).

### POS Bind Toll-Like Receptor

The oral administration of POS reduced the incidence of diarrhea and DAI, which shortens the length of colon caused by DSS. Importantly, it was found that POS showed an anti-colitis effect that appears to be related to its ability to downregulate COX-2 of TLR4/NF-κB pathway (129, 139). POS administration affects the activation of TLR4/NF-κB/COX-2 signaling cascade by binding TLR (Figure 6) (140). The level of TLR4 is associated with cardiac disorders and regarded as a clinical biomarker of heart disease (141).

COX-2 expression is closely related to TLR4/NF-κB pathway in the intestine, particularly in the setting of DSS colitis. As a key receptor in innate immunity, TLR4 has been found to be overexpressed in UC patients (142, 143). TLR4-modulated signaling further activates NF-κB, which is followed by expression of an array of subsequent genes participating in inflammatory signaling cascades that mediate the pathogenesis of colitis (Figure 6).

Understanding UC pathogenesis and progress has greatly accelerated the discovery of many therapeutic drugs targeting targeted inflammatory signaling, such as TLR4/NF-κB/COX-2 signaling pathway (Figure 6). COX-2 contributes to the production of inflammatory mediators of PGE2 (144). Consistent with the results of the POS anti-inflammatory mechanism obtained in other diseases, POS has been found to significantly downregulate COX-2 expression (145). Many therapeutic agents have been considered to eliminate intestinal inflammation in UC by blocking TLR4/NF-κB pathways. TLR4 is highly expressed in inflammatory mucosa of UC patients. As a pattern recognition receptor, TLR4 plays a key role in preventing intestinal pathogens. However, since TLR4 is considered to be the most important inflammatory inducer of all members of the TLR family, TLR4-mediated inflammation-related intestinal dysfunction further contributes to the development of UC. NF-κB can be stimulated by TLR4, which is a key transcription factor for inducing and regulating a series of inflammatory mediators. Apple POS has been found to significantly reduce the protein levels of TLR4 and NF-κB, suggesting that inhibition of TLR4/NF-κB pathway and its downstream COX-2 is associated with anti-inflammation properties of POS (Figure 6).

### Controversies of the Present Review

**Figure 7:**
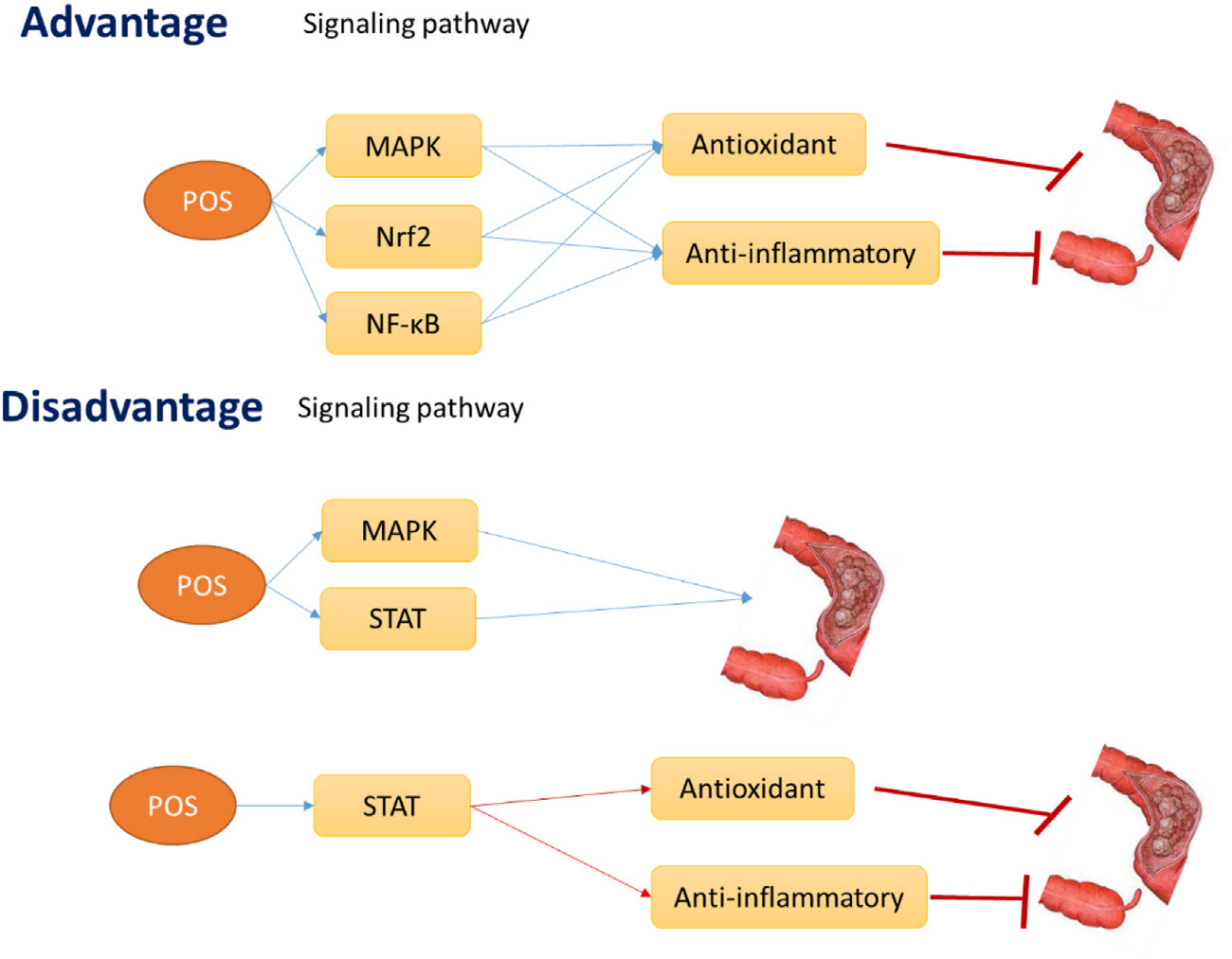
The hypothesis of the advantage and disadvantage effects of pectin oligosaccharides on colon cancer risk.

The review focuses on antioxidant and anti-inflammatory roles of POS for promoting human health by regulating some potential oxidative and inflammation-activated ATP-activated protein kinase (AMPK), nuclear factor erythroid-2-related factor-2 (Nrf2), and NF-κB pathways. The activation of these signaling pathways increases the antioxidant and anti-inflammatory activities, which will result in the apoptosis of CC cells or in the prevention of CC risk and progression. Thus, POS may inhibit CC development by affecting antioxidant and anti-inflammatory signaling pathways AMPK, Nrf2, and NF-kB (Figure 7). However, POS also can activate STAT1 and 3 signaling pathway, which will reduce antioxidant and anti-inflammatory properties and promote CC progression (Figure 7). Furthermore, activation of AMPK and STAT also can promote CC progression (Figure 7). STAT signaling pathway also inhibits antioxidant and anti-inflammatory activities. All the results will be converse to the widely accepted antitumor properties of POS.

## Conclusion

The present review provides signaling-pathway molecular mechanism of POS (the degraded products of pectin), which is different current widely accepted thoughts for pectin: pectin as a dietary fiber (146), pH-modified pectin (42), modified pectin to avoid chemoresistance (147), and pectin as a drug delivery system in tumor therapy (148).

Anti-inflammatory and antitumor effects for CC of natural products have been widely explored and studied. It is highly demanded for bio-materials used in functional food with few side effects and environmentally friendly properties. POS, as soluble dietary fibers with various health-promoting functions, have a good potential in controlling oxidant stress and inflammatory situation by affecting antioxidant and anti-inflammatory mediated signaling pathways, which contribute to antitumor effects on CC. Structurally modified POS may be associated with their functions and should be carefully selected. The present review paper lacks the important information for the linkage between the specific structure of POS and its function. To further understand the prebiotic role of POS and their derivatives effects on antitumor therapy, the specific POS with a certain degree of polymerization or purified polymers are highly demanded to be performed in clinical practice.

## Author Contributions

HT collected all the literature. WC, GY, and QL analyzed all these data. KL wrote the paper.

## Conflict of Interest Statement

The authors declare that the research was conducted in the absence of any commercial or financial relationships that could be construed as a potential conflict of interest.
